# Targeting *ERBB2* (*HER2*) Amplification Identified by Next-Generation Sequencing in Patients With Advanced or Metastatic Solid Tumors Beyond Conventional Indications

**DOI:** 10.1200/PO.18.00345

**Published:** 2019-10-21

**Authors:** Ecaterina E. Ileana Dumbrava, Kavitha Balaji, Kanwal Raghav, Kenneth Hess, Milind Javle, Mariela Blum-Murphy, Jaffer Ajani, Scott Kopetz, Russell Broaddus, Mark Routbort, Mehmet Demirhan, Xiaofeng Zheng, Shubham Pant, Apostolia M. Tsimberidou, Vivek Subbiah, David S. Hong, Jordi Rodon, Kenna M. Shaw, Sarina A. Piha-Paul, Funda Meric-Bernstam

**Affiliations:** ^1^ The University of Texas MD Anderson Cancer Center, Houston, TX; ^2^ Lexicon Pharmaceuticals, Houston, TX

## Abstract

**PURPOSE:**

Human epidermal growth factor receptor 2 (HER2) is an effective therapeutic target in breast and gastric and gastroesophageal junction cancers. However, less is known about the prevalence of *ERBB2* (*HER2*) amplification and the efficacy of HER2-targeted treatment in other tumors.

**PATIENTS AND METHODS:**

We assessed *HER2* amplification status among 5,002 patients with advanced disease (excluding breast cancer) who underwent next-generation sequencing. We evaluated the clinical benefit of HER2-targeted therapy by measuring the time-dependent overall survival (OS) from the genomic testing results, progression-free survival (PFS), and PFS during HER2-targeted therapy (PFS2) compared with PFS during prior therapy (PFS1).

**RESULTS:**

Overall, 122 patients (2.4%) had *HER2* amplification, including patients with endometrial (5.3%), bladder (5.2%), biliary or gallbladder (4.9%), salivary (4.7%), and colorectal cancer (3.6%). Forty patients (38%) with nongastric, nongastroesophageal junction, or nonesophageal cancers received at least one line of HER2-targeted therapy. Patients receiving HER2-targeted therapy had a median OS of 18.6 months, compared with 10.9 months for patients who did not receive HER2-targeted therapy (*P* = .070). On multivariable analysis, HER2-targeted therapy was significantly associated with increased OS (hazard ratio, 0.5; 95% CI, 0.27 to 0.93; *P* = .029), regardless of sex, age, or number of prior lines of treatment. The PFS2-to-PFS1 ratio was 1.3 or greater in 21 (57%) of 37 patients who received HER2-targeted therapy not in the first line of systemic treatment, and the median PFS2 and PFS1 times were 24 and 13 weeks, respectively (*P* < .001).

**CONCLUSION:**

*HER2* amplifications using next-generation sequencing can be identified in a variety of tumor types. HER2-targeted therapy may confer clinical benefit in tumor types other than those for which HER2 inhibitors are approved.

## INTRODUCTION

Personalized cancer therapy is becoming more histology agnostic as treatments are chosen based on tumor genomics rather than tumor type.^1,2^ Human epidermal growth factor receptor 2 (HER2) is a transmembrane tyrosine kinase receptor that belongs to the epidermal growth factor receptor family and is encoded by the *ERBB2* (*HER2*) gene (chromosome 17q12). Overexpression of HER2 protein occurs through *HER2* gene amplification or through other transcriptional or translational mechanisms^3^ and can result in the formation of spontaneous receptor homodimers, resulting in the initiation of downstream signaling cascades and malignant transformation.^4,5^ *HER2* amplification is a prognostic biomarker for worse survival in the absence of anti-HER2 therapy.^6,7^

HER2 is a compelling therapeutic target in patients with breast^6,8-10^ and gastric or gastroesophageal junction (GEJ) cancers.^11^ For HER2-overexpressing or HER2-amplified breast cancer, several HER2-targeted therapies are approved for use in the adjuvant and metastatic settings, including trastuzumab (metastatic and adjuvant), pertuzumab (metastatic and adjuvant), lapatinib (metastatic), ado-trastuzumab emtansine (metastatic), and neratinib (adjuvant). Trastuzumab is also approved, in combination with cisplatin and a fluoropyrimidine (capecitabine or fluorouracil), for the treatment of metastatic gastric or GEJ cancers. Furthermore, several promising novel HER2-targeted agents are in development, such as the bispecific HER2 antibody ZW25 and the antibody-drug conjugate DS-8201.^12,13^

**CONTEXT**

**Key Objective**
Our study focused on assessment of *ERBB2* (*HER2*) amplification in patients with solid tumors, excluding breast cancer, who underwent next-generation sequencing. We evaluated the clinical benefit of HER2-targeted therapy by measuring the time-dependent overall survival from the genomic testing results, progression-free survival (PFS), and PFS during HER2-targeted therapy compared with PFS during prior therapy.
**Knowledge Generated**
We showed that *HER2* amplification is present in a clinically relevant proportion of tumors and in a variety of tumor types and that HER2-targeted therapy may confer clinical benefit, with increased survival in patients with tumor types other than those for which HER2 inhibitors are approved.
**Relevance**
HER2 is an established effective therapeutic target in breast, gastric, and gastroesophageal junction cancers; however, less is known about the prevalence of *HER2* amplification and efficacy of HER2-targeted treatment in other tumors. The results showed *HER2* amplifications in patients with various tumor types, including endometrial (5.3%), bladder (5.2%), biliary or gallbladder (4.9%), salivary (4.7%), and colorectal cancer (3.6%). Patients who received matched HER2-targeted therapies had significantly increased PFS on HER2-targeted therapy compared with previous treatment and increased overall survival. Validation of these results in a larger study could focus on determining the associations of copy number, simultaneous *HER2* mutations, and other coalterations with response to HER2-targeted therapies.

The main mechanism of HER2 overexpression is *HER2* gene amplification, which occurs in 18% to 20% of patients with breast cancer^14,15^ and 7% to 34% of patients with gastric or GEJ cancers.^11,16,17^ ASCO and the College of American Pathologists recommended testing in breast and gastric or GEJ cancers using immunohistochemistry (IHC) for HER2 protein expression or in situ hybridization (fluorescence in situ hybridization [FISH], chromogenic in situ hybridization, or silver in situ hybridization) for *HER2* gene amplification.^14,18^ Other techniques, such as comparative genomic hybridization, can also be used to detect copy number variations.^19-21^ However, with the development and integration of next-generation sequencing (NGS) in cancer care and the increasing capacity of NGS to determine copy number variations concurrently with other alterations such as mutations, NGS has become a more cost-effective and tissue-efficient alternative to current single-gene assessment methods.^22^

*HER2* amplification also occurs in other carcinomas at differing frequency.^23-25^ Although relatively little is known about the role of HER2 in other tumor types, emerging data indicate that HER2-targeted therapy may have efficacy in other HER2-positive tumors.^26^

We hypothesized that HER2-targeted therapy could be associated with clinical benefit in tumor types other than breast and gastric or GEJ cancers. To test this hypothesis, we determined the prevalence of *HER2* amplification determined by NGS in different tumor types and compared progression-free survival (PFS) during matched HER2-targeted therapy with PFS during prior therapy. We also compared the overall survival (OS) of patients who received HER2-targeted therapy with the OS of patients who did not.

## PATIENTS AND METHODS

### Selection of Patients

Patients with advanced or metastatic solid tumors (excluding breast cancer and lymphoma) underwent NGS in Clinical Laboratory Improvement Amendments–certified laboratories using multiple platforms to facilitate personalized cancer therapy between January 2011 and June 2017. For the current study, the NGS analysis was performed using four platforms, including the Oncomine Comprehensive Assay (ThermoFisher, Waltham, MA) or Ion AmpliSeq Comprehensive Cancer Panels (ThermoFisher) performed at The University of Texas MD Anderson Cancer Center Molecular Diagnostic Laboratory,^27^ FoundationOne or FoundationOne Heme (Foundation Medicine, Cambridge, MA) tumor testing, or Guardant360 (Guardant Health, Redwood City, CA) circulating cell-free DNA (cfDNA) testing. We excluded patients in whom *HER2* amplification was detected by NGS on platforms that do not systematically report copy number variation. The genomic testing results were annotated by the Precision Oncology Decision Support System at The University of Texas MD Anderson Cancer Center.^28^

The patients’ relevant clinical and molecular characteristics were collected from electronic medical records and prospectively maintained institutional databases (Table 1). The diagnosis was obtained from the pathology reports that had been verified by board-certified pathologists at The University of Texas MD Anderson Cancer Center. Other profiling, such as IHC for HER2 protein expression and FISH for *HER2* amplification, was performed in some patients and was also reviewed in this study.

**TABLE 1.**
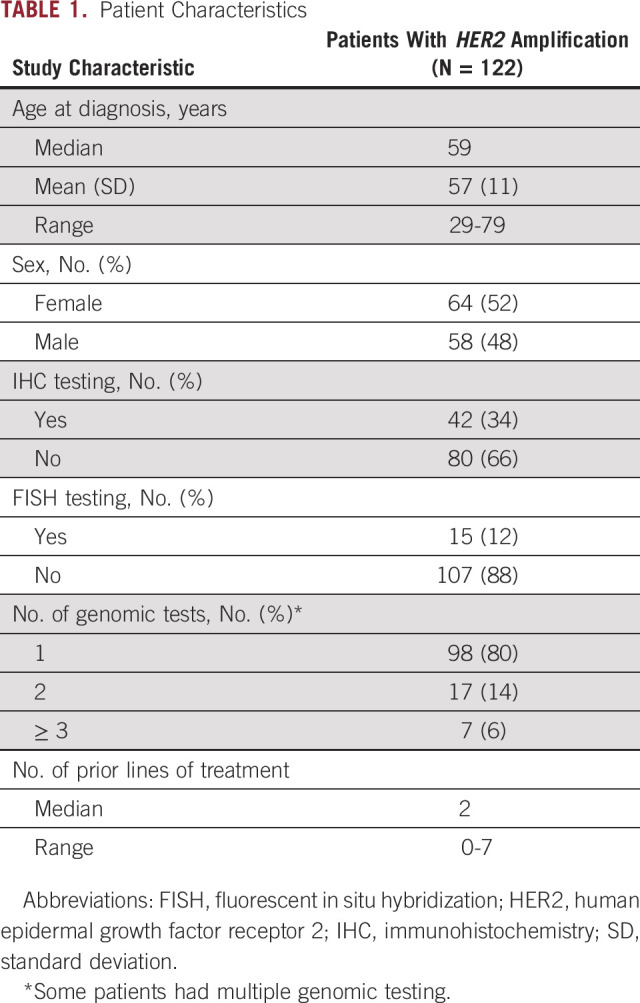
Patient Characteristics

The HER2-targeted clinical trials had been individually approved and conducted at The University of Texas MD Anderson Cancer Center in accordance with institutional review board guidelines, and this reported analysis was conducted under an institutional review board–approved protocol.

### *HER2* Amplification and Overexpression Analysis

*HER2* amplification determined by NGS was defined according to each platform’s analytic pipeline, was based on the resulting reports and validation, and varied between greater than five to greater than seven estimated copy numbers for reporting high-confidence amplification.^29,30^ For patients who underwent cfDNA analysis, digital sequencing was performed by Guardant Health, using a 54-gene panel (Guardant360). *HER2* plasma copy numbers of 2.5 to 4.0 are reported as ++ amplification, and greater than 4.0 copy numbers are reported as +++ amplification, representing the 50th to 90th and greater than 90th percentiles, respectively, of all copy number alteration calls in the Guardant360 database.^31^ IHC staining for HER2-neu and *HER2* FISH analysis were performed on specimens from some patients with *HER2* amplification (Appendix).

### Clinical Benefit on HER2-Targeted Therapy

We investigated the anticancer treatments received by patients with *HER2* amplifications. To determine the clinical benefit of HER2-targeted therapy, we measured PFS during matched HER2-targeted therapy (PFS2) and compared it with PFS during prior therapy (PFS1).^32,33^ PFS was defined as the time from the start of treatment until disease progression or death. Response to treatment and progression were determined using Response Evaluation Criteria in Solid Tumors (RECIST) version 1.1, as measured by radiologists or investigators.^34^ Patients who received HER2-targeted therapy as the first systemic treatment were excluded from the PFS2-to-PFS1 analysis.

We also evaluated the OS of patients who received HER2-targeted therapy and compared it with the OS of patients who did not receive HER2-targeted therapy. OS was calculated as a time-dependent indicator variable in both the Kaplan-Meier and Cox proportional hazards analyses from the genomic testing result until death from any cause. Last news and death date were determined based on the electronic medical records, and survival follow-up was updated in March 2019. The Royal Marsden Hospital prognostic score for predicting survival in phase I trials^35^ (including albumin, lactate dehydrogenase [LDH], and number of metastatic sites), number of prior lines of treatment, disease stage, and Eastern Cooperative Oncology Group (ECOG) performance status at the time of genomic testing were also analyzed.

### Statistical Analysis

We used descriptive statistics to summarize the characteristics of patients with *HER2* amplifications. Concordance between NGS and IHC and between NGS and FISH tests was calculated by dividing the number of samples that had concordant results by the total number of samples.

Univariable and multivariable Cox proportional hazards models were fit to assess the association between prognostic factors and OS, in which the prognostic factors included HER2-targeted therapy, sex, age, histology, ECOG performance status, number of prior therapies, number of metastatic sites, disease stage, LDH, albumin, and number of metastatic sites at time of genomic testing. All statistical analyses were carried out using SPSS version 24 (SPSS, Chicago, IL), Prism 7 (Graphpad, San Diego, CA), or RStudio (https://www.rstudio.com/).

## RESULTS

### Prevalence of *HER2* Amplification

**FIG 1.**
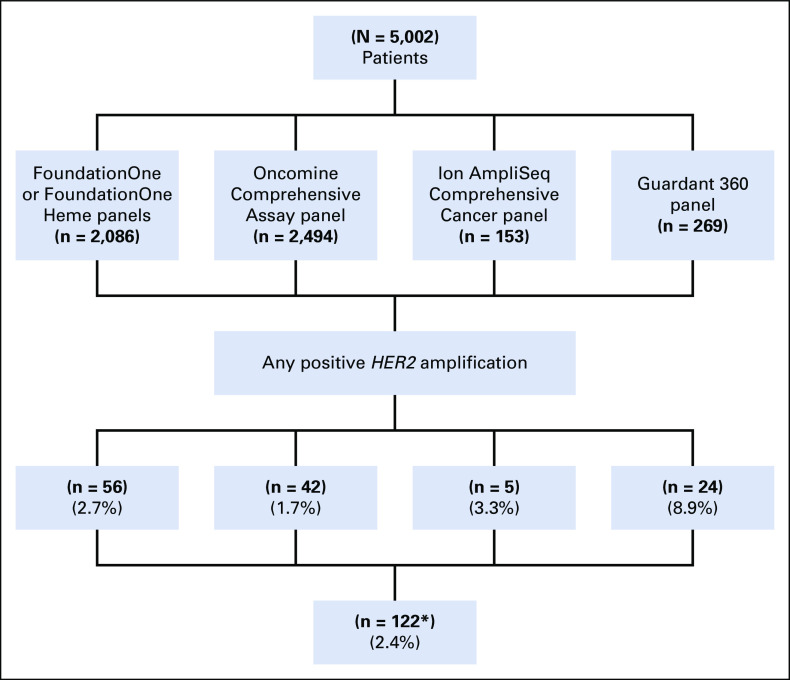
CONSORT diagram for patient selection by next-generation sequencing results. (*) Some patients had multiple genomic testing. HER2, human epidermal growth factor receptor 2.

A total of 5,002 patients with advanced solid tumors met our eligibility criteria. *HER2* amplification was found by NGS in 122 patients (2.4%). All patients with *HER2* amplifications had advanced or metastatic solid tumors and had received an average of two prior lines of treatment before the genomic testing. One hundred six patients were found to have *HER2* amplification on tumor tissue analysis on the FoundationOne, Oncomine Comprehensive Assay, or Ion Torrent AmpliSeq Comprehensive Cancer platforms, and 24 patients were found to have *HER2* amplifications on cfDNA analysis using Guardant360 technology (Fig 1). Ten patients had testing on more than one panel.

**FIG 2.**
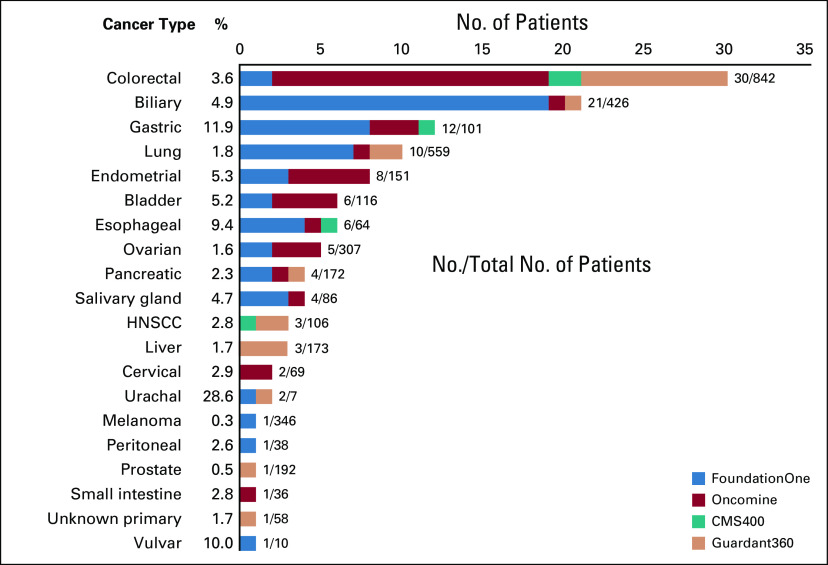
Prevalence of *HER2* amplification. CMS400, Ion AmpliSeq Comprehensive Cancer panel; HER2, human epidermal growth factor receptor 2; HNSCC, head and neck squamous cell carcinoma; Oncomine, Oncomine Comprehensive Assay panel.

The frequency of *HER2* amplifications identified by NGS (in tumor types with > 10 patients) ranged from 0.3% in melanoma to 11.9% in gastric or GEJ cancers. The most frequent *HER2*-amplified tumor types included gastric or GEJ, esophageal, endometrial, bladder, biliary or gallbladder, salivary gland, colorectal, and cervical tumors (Fig 2). In contrast, no *HER2* amplification was detected by NGS in 382 patients with sarcomas, 224 patients with glioblastomas, 132 patients with thyroid cancers, 97 patients with renal cell carcinomas, 66 patients with neuroendocrine tumors, 50 patients with lymphoma, and 37 patients with appendiceal carcinoma.

### Concordance of *HER2* Amplification Between NGS, IHC, and FISH

NGS was performed on two or more platforms in 24 patients (20%), with seven patients having three or more NGS tests. We compared the concordance of the results of *HER2* amplification and HER2 protein expression.

Forty-two (34%) of 122 patients with *HER2* amplifications on NGS also underwent HER2 IHC testing. Of these 42 patients, 31 (74%) had HER2 protein overexpression (3+), four (9%) had equivocal expression (2+), two (5%) had low expression (1+), and five (12%) had no HER2 expression. Among the seven patients who were found to have low or no HER2 expression on IHC, three had equivocal *HER2* amplification on NGS and two had 1+ *HER2* amplification on cfDNA testing

Sixteen patients had testing by FISH in addition to NGS. Fourteen FISH results (88%) were concordant with the NGS result for *HER2* amplification. Of the two patients with discordant results, one had low-level (1+) *HER2* amplification on cfDNA, and the other had equivocal amplification on FoundationOne.

Twenty-four patients had positive *HER2* amplification on the Guardant360 platform for cfDNA. Of these, 11 patients (46%) had a strong (2+, n = 6) or very strong (3+, n = 5) positive result. Among these 11 patients, five also had IHC testing, all with concordant positive HER2 protein expression, and *HER2* amplification was confirmed in all three patients who had FISH testing (Appendix Table A1).

### Clinical Benefit of HER2-Targeted Therapy

We studied the clinical actionability of *HER2* amplification and clinical benefit of HER2-targeted therapy in 122 evaluable patients who had the molecular testing done more than 6 weeks from the current analysis. Response to treatment was determined by RECIST version 1.1, except in three patients who had clinical progression without radiologic documentation of progressive disease.

Forty patients with other tumor types than the ones for which HER2 inhibitors are approved (38%) received at least one line of HER2-targeted therapy, with eight patients receiving more than one line of HER2-targeted therapy. Most patients (93%) received trastuzumab in combination with chemotherapy or other targeted therapies. Across different lines of treatment, 27 patients received trastuzumab with other targeted therapies such as pertuzumab, 14 patients received trastuzumab and chemotherapy, three patients received small-molecule inhibitors targeting HER2, three patients received antibody-drug conjugates or bispecific antibodies against HER2, and two patients received trastuzumab alone. For three patients with nongastric or non-GEJ cancers, the HER2-targeted therapy was the first line of treatment.

**FIG 3.**
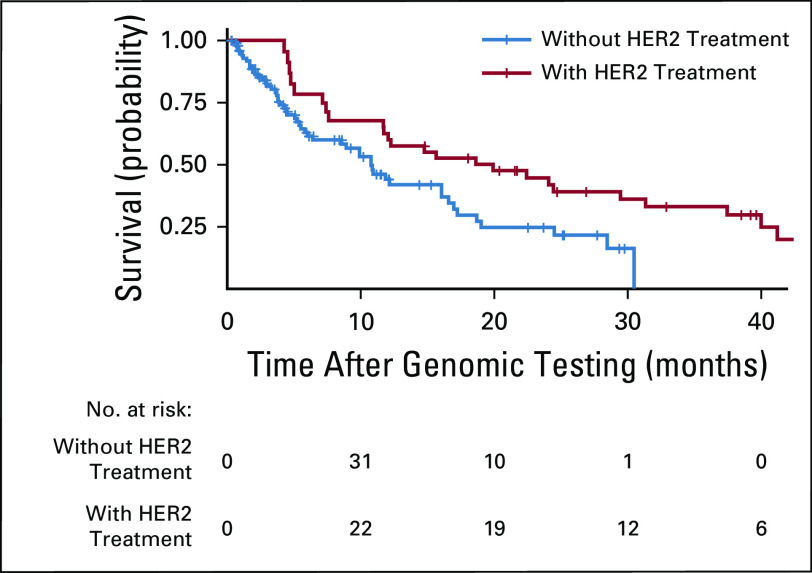
Overall survival (OS) of patients with human epidermal growth factor receptor 2 (*HER2*) amplification, excluding patients with gastric or gastroesophageal junction cancers. HR, hazard ratio.

After the exclusion of patients with gastric, GEJ, or esophageal cancers, patients receiving HER2-targeted therapy had a longer median OS than patients who did not receive such therapy (18.6 and 10.9 months, respectively; hazard ratio, 0.60; 95% CI, 0.34 to 1.06; *P* = .07; Fig 3). Receiving HER2-targeted therapy was associated with improved OS in multivariable Cox proportional hazards analysis in patients without gastric, GEJ, or esophageal cancers (hazard ratio, 0.50; 95% CI, 0.27 to 0.93). Other factors associated with a longer OS were ECOG performance status of 0 or 1, a Royal Marsden Hospital prognostic score of 0 or 1 (normal albumin and LDH levels and two or fewer metastatic sites), and colorectal cancer tumor type as compared with other histologies (Table 2).

**TABLE 2.**
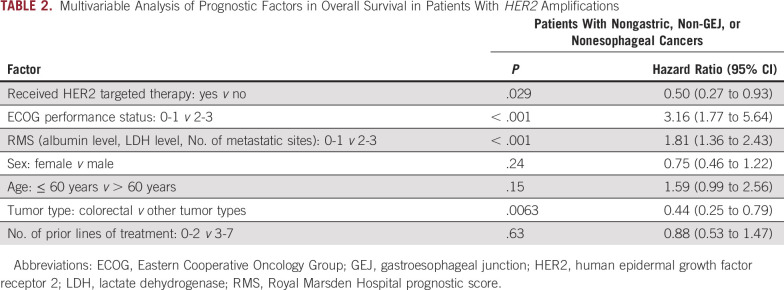
Multivariable Analysis of Prognostic Factors in Overall Survival in Patients With *HER2* Amplifications

**FIG 4.**
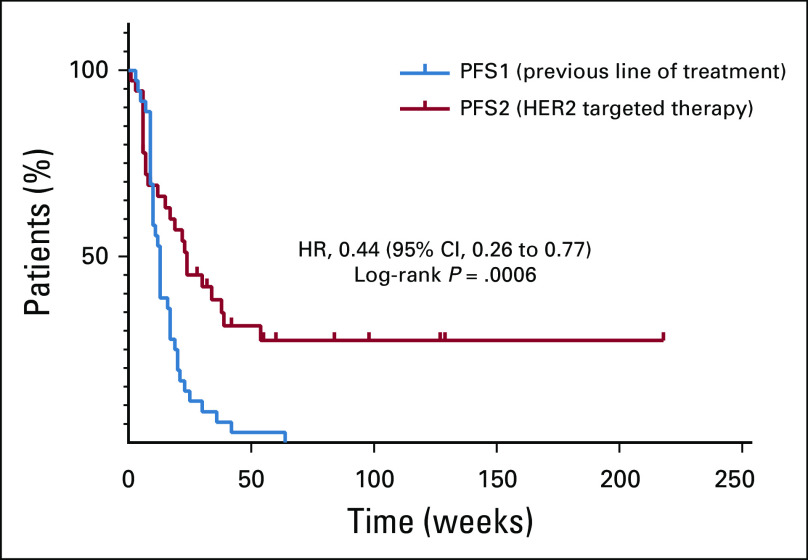
Progression-free survival (PFS) of patients with human epidermal growth factor receptor 2 (*HER2*)–amplification, excluding patients with gastric or gastroesophageal junction cancers. PFS during HER2-targeted therapy (PFS2) compared with PFS during prior therapy (PFS1). HR, hazard ratio.

Among 37 patients with cancers other than gastric, GEJ, or esophageal cancers in whom HER2-targeted treatment was given in the second line or later and for whom previous treatment information was available, the PFS2-to-PFS1 ratio was 1.3 or greater in 21 patients (57%), and the median PFS2 and PFS1 times were 24 and 13 weeks, respectively (*P* < .001; Fig 4).

Twelve (30%) of 40 patients with tumor types other than gastric cancer achieved an objective response as defined by complete or partial response per RECIST version 1.1, with seven patients receiving trastuzumab and pertuzumab, four patients receiving trastuzumab with chemotherapy, and one patient receiving an HER2 antibody-drug conjugate. In addition, nine patients had stable disease per RECIST version 1.1 for at least 24 weeks.

### Determinants of Enrollment on HER2-Targeted Therapy

**FIG A1.**
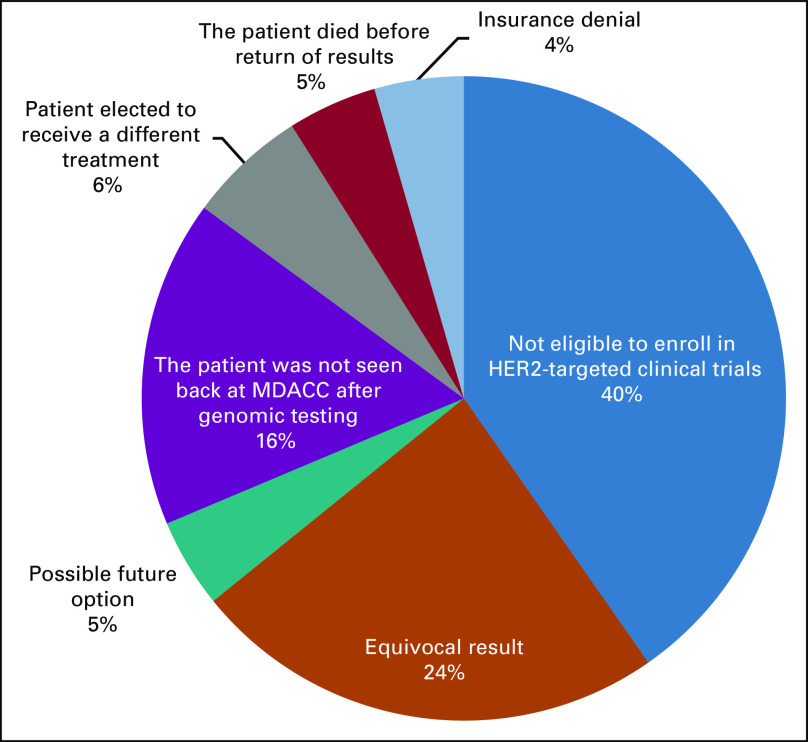
Determinants of enrollment in human epidermal growth factor receptor (HER2)–targeted therapy trials. MDACC, MD Anderson Cancer Center.

The median time between NGS showing an *HER2* amplification and start of HER2-targeted therapy was 16 weeks. We investigated the reasons why 68 patients did not receive HER2-targeted therapy after their NGS results showed *HER2* amplification. The leading cause was noneligibility for an HER2-targeted clinical trial (n = 28; 41%) because of equivocal *HER2* amplification results, insurance denial, or clinical issues such as poor performance status, chronic tumor-related bleeding, or inadequate organ function (Appendix Fig A1).

## DISCUSSION

In a large cohort of patients who underwent targeted NGS to facilitate personalized cancer treatment, we found *HER2* amplification in tumor types other than breast and gastric or GEJ cancers. HER2-matched targeted therapy in patients with tumor types other than those for which HER2 inhibitors are approved was associated with a clinically significant increase in OS but with only a trend toward a statistically significant increase (*P* = .070).

Until recently, almost all studies of HER2 status focused on one type of malignancy, making it difficult to compare the rate of HER2 positivity across studies and tumor types.^36-40^ Furthermore, HER2 overexpression or amplification is most often evaluated by IHC or FISH, rather than NGS.^41,42^ However, in the current era of personalized cancer therapy, NGS is becoming more widely used. NGS has been shown to meet the sensitivity of detection for mutations used in clinical trials, permitting simultaneous testing of copy number variations in hundreds of genes.^28,40,43^

Currently, there are several ongoing clinical trials evaluating prevalence of *HER2* alterations and the benefit of targeting HER2 in different tumor types (eg, ClinicalTrials.gov identifiers: NCT02465060, NCT02675829, NCT02091141, NCT02693535). The recently published results from the MyPathway trial^26,45^ studying treatment with trastuzumab and pertuzumab in colorectal cancer showed an overall response rate of 40% in patients without *KRAS* mutations, confirming preliminary data that HER2 testing could be integrated in future guidelines for biomarker testing in other tumor types, such as colorectal,^44,46^ salivary, bladder, and biliary cancers. On the basis of these findings, the National Comprehensive Cancer Network colorectal cancer guidelines were updated recently to include pertuzumab plus trastuzumab and trastuzumab plus lapatinib as category 2B recommendations for HER2-positive colorectal cancer.^47^

In this study, NGS identified *HER2* amplification in 2.4% of patients across 20 tumor types. Although *HER2* amplifications were found in many different epithelial cancers, positive results were rare, and often nonexistent, in malignancies of nonepithelial origin. This finding was consistent with the HER2 overexpression results reported by Yan et al^42^ in 37,992 patients. Furthermore, our frequency of *HER2* amplification in colorectal cancer (3.6%) is consistent with a previously reported prevalence between 2% and 6%.^48^

In contrast with breast cancer, for which HER2-targeted therapies have been established for a long time with five treatment options approved by the US Food and Drug Administration, for gastric, GEJ, or esophageal cancers, less is known about the prognostic role of HER2, and therapeutic options are limited to trastuzumab in combination with chemotherapy.^26^ Our results suggest there is a clinical benefit in patients with indications beyond gastric or GEJ cancers.

In tissue samples, the thresholds for reporting are higher for NGS than for FISH; therefore, the tissue-based NGS test used in our study may have underestimated the rate of *HER2* amplification. In contrast, we also included cfDNA testing, in which amplification of 1+ corresponds to less than 2.4 copy numbers. Furthermore, the patient population referred for genomic testing and consideration for participation in clinical trials might be different from the overall population. Our results on testing for HER2 status by NGS compared with IHC and FISH are consistent with a previous report of high concordance between IHC and FISH in colorectal cancer.^49,50^

Many patients were not eligible for HER2-targeted therapies, highlighting the importance of patient selection for genomic testing. However, as evidence for actionability of HER2 increases, HER2 testing should be considered earlier in the treatment course for tumor types in which *HER2* is more frequently amplified (eg, colorectal cancer).

Sequential testing by IHC and FISH and further mutation analyses may lead to tissue exhaustion before the completion of all necessary testing. Thus, early incorporation of NGS into clinical practice for diseases with frequent actionable genomic alterations has the advantage of screening for multiple therapeutic options simultaneously while sparing tissue.

Our study has several limitations that might limit the generalizability of our findings. Our cohort was heterogeneous, and a limited number of patients were treated in clinical trials with strict eligibility criteria, making conclusive determinations problematic. Although NGS has many advantages, samples with low tumor content, heterogeneity, and low levels of amplification may result in false-negative results where *HER2* amplification might have been detected on FISH^20^; thus, we are likely underestimating the frequency of *HER2* amplification.^51^ A higher prevalence of *HER2* amplification on liquid biopsies could be, at least in part, related to a selection bias and may be consistent with emergence of *HER2* amplification as a mechanism of resistance to epidermal growth factor receptor–targeted therapy,^52-54^ explaining the higher discordance rates when compared with gold standard tissue-based tests. However, patients with *HER2* amplification detected on NGS may have higher levels of amplification and therefore may have greater benefit from HER2-targeted therapy.

NGS reveals *HER2* amplification in a clinically relevant proportion of tumors and in a variety of tumor types, and HER2-targeted therapy may confer clinical benefit in tumor types beyond those for which HER2 inhibitors are approved. Our results showed an increased survival with matched HER2-targeted therapies in patients with *HER2* amplifications. Further studies are needed to confirm these results and to determine the associations of copy number, simultaneous *HER2* mutations, and other coalterations with response to HER2-targeted therapies. The association of *HER2* amplifications with genomic alterations in other oncogenic drivers provides rationale for novel therapeutic combinations.

## Figures and Tables

**TABLE A1.**
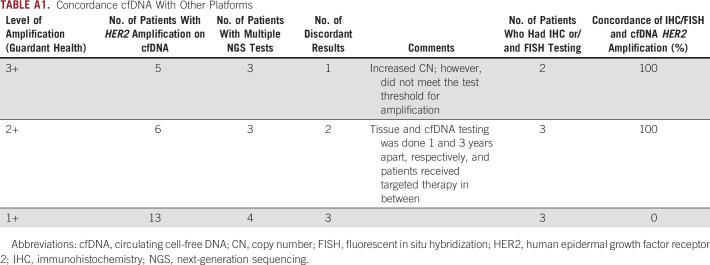
Concordance cfDNA With Other Platforms

## Appendix

### *ERBB2* Amplification by Circulating Cell-Free DNA Analysis (Guardant360)

Human epidermal growth factor receptor 2 (*HER2*) plasma copy numbers of 2.5 to 4.0 are reported as ++ amplification, and copy numbers greater than 4.0 are reported as +++ amplification, representing the 50th to 90th and greater than 90th percentiles, respectively, of all copy number alteration calls in the Guardant360 (Guardant Health, Redwood City, CA) database.

### HER2 Overexpression by Immunohistochemistry

HER2 overexpression was defined as strong, complete, homogenous membrane staining in more than 30% of invasive tumor cells (score, 3+). Negative results were defined as those with no staining (score, 0) or faint or barely perceptible membranous staining (score, 1+) in less than 10% of the invasive tumor cells. Equivocal results were defined as those with weak to moderate complete, basolateral, or lateral membranous reactivity in at least 10% of invasive tumor cells (score, 2+).

### *HER2* Amplification by Fluorescent In Situ Hybridization

*HER2* fluorescent in situ hybridization was also performed in some patients, and *HER2* amplification was defined as an overall ratio of 2.0 or greater with an average *HER2* copy number of greater than 4.0 signals per cell; an overall ratio of 2.0 or greater with an average *HER2* copy number of less than 4.0 signals per cell; an average ratio of 2.0 or greater with an average *HER2* copy number of 4.0 or more but less than 6 signals per cell; or an average ratio of less than 2.0 with an average *HER2* copy number of 6.0 or more signals per cell.
